# Accuracy and limitations of equations for predicting the glomerular filtration rate during follow-up of patients with non-diabetic nephropathies

**DOI:** 10.1186/1471-2369-10-16

**Published:** 2009-06-25

**Authors:** Guy Rostoker, Pierre Andrivet, Isabelle Pham, Mireille Griuncelli, Serge Adnot

**Affiliations:** 1 Service de Néphrologie et de Dialyse, Centre Hospitalier Privé Claude Galien, 20 route de Boussy Saint Antoine, 91480 Quincy sous Sénart, France; 2 Service de Physiologie et d'explorations fonctionnelles, Centre Hospitalier Universitaire Henri Mondor, 51, Avenue du Maréchal de Lattre de Tassigny, 94000 Créteil, France

## Background

Rapid and accurate estimation of the glomerular filtation rate (GFR) is required for many major clinical decisions in patients with chronic nephropathies [1]. Direct GFR measurement is time-consuming and expensive, frequently requires urine collection and isotope use, and is routinely available in only a few medical centers [1]. In clinical practice, GFR is usually estimated from the serum creatinine concentration. However, this last is affected by factors other than creatinine glomerular filtration, such as diet, muscle mass, tubular secretion, unstable renal function, colorimetric interference, and day-to-day assay variability [1]. To circumvent these limitations, several equations have been developed to estimate GFR from the serum creatinine concentration adjusted for age, sex, body weight and demographic factors [1]. The equation proposed by Cockcroft and Gault in 1976 is widely used throughout the world [2,3]. Adjustment for body surface area has been shown to improve the accuracy of the original Cockcroft-Gault equation [4]. In recent years, the Modification of Diet in Renal Disease (MDRD) group developed three multiple regression models that improved the prediction of GFR from the plasma creatinine concentration [5]. The first includes urinary urea excretion and the second is derived from demographic factors combined with serum creatinine, urea and albumin; the third, which is slightly less accurate, uses demographic factors and serum creatinine (MDRD abbreviated equation) [5]. Finally, the Mayo Clinic team have developed a quadratic equation (MCQ) based on results of both healthy subjects and patients with chronic renal diseases [6].

The validity of these creatinine-based equations for the follow-up of renal function in patients with known renal disease is uncertain, notably during therapeutic interventions and data on this topic are scarce [7]. Indeed, five studies, all restricted to patients with diabetic nephropathy suggested that, in patients with normal renal function or hyperfiltration (microalbuminuric), prediction equations are not accurate enough to monitor kidney function, whereas in chronic kidney disease (CKD) stages 2 and 3 these equations may be valid [8-12]. The aim of this study was therefore to compare the accuracy of prediction equations (original Cockcroft and Gault equation, Cockcroft and Gault equation adjusted for body surface area, Abbreviated MDRD and Mayo Clinic Quadratic Equation) for the follow-up of non-diabetic nephropathies, by comparison with inulin clearance, the gold standard for GFR estimation. We analyzed data from a prospective cohort of 260 European patients with non-diabetic chronic kidney disease [4], 126 of whom had repeated measures of their GFR based on inulin clearance during their long-term follow-up.

## Methods

### Study population

We recently reported a cross-sectional investigation in which we prospectively studied 269 European adults with chronic renal disease (260 with non diabetic nephropathies and 9 with diabetes mellitus) [4]. We also conducted a longitudinally study of 126 patients of this cohort. These patients had non-diabetic nephropahies (mainly glomerular diseases) and had repeated measures of their GFR by inulin clearance during long-term follow-up. A total of 452 inulin clearance assays were carried out in these patients. All the patients gave their informed consent to the study, which was approved by the local ethics committee.

Basing on inulin clearance, the patients were divided into two subgroups: patients whose renal function deteriorated during follow-up (n = 65) and patients whose renal function improved (n = 61).

The subgroup of patients whose renal function deteriorated during follow-up comprised 51 men and 14 with a median age of 37 years (range 20–65 y). Twenty-four patients had primary IgA nephropathy, 13 had idiopathic membranous nephropathy, 7 had an idiopathic nephrotic syndrome, 3 had mesangioproliferative glomerulonephritis, 9 had systemic diseases (Henoch-Schönlein purpura: 4; lupus erythematosus: 2; primary sicca syndrome: 2; ANCA vasculitis:1), 5 had hereditary nephritis (1 polycystic renal disease, 1 Alport syndrome; 1 sickle cell anemia, 2 miscellaneous) and 4 had inherited or acquired urologic abnormalities of the kidneys, with no repercussions on bladder voiding.

The subgroup of patients whose renal function improved during follow-up comprised 39 men and 22 women with a median age of 43 years (range 17–69 y). Nineteen patients had primary IgA nephropathy, 9 had idiopathic membranous nephropathy, 11 had an idiopathic nephrotic syndrome, 2 had mesangioproliferative glomerulonephritis, 2 had crescentic glomerulonephritis, 7 had systemic diseases (lupus erythematosus: 4; Behçet disease: 1; systemic sclerosis: 1, Atkinson-Clarkson syndrome 1), 2 had hypertensive nephritis (nephroangiosclerosis), 3 had hereditary nephritis (1 polycystic renal disease, 1 thin glomerular membrane disease, 1 Fabry disease) and 6 had inherited or acquired urologic abnormalities of the kidneys with no repercussions on bladder voiding.

### GFR measurements

GFR was measured in each patient by using the reference inulin method (GFR-inulin) at the Physiology Department of Henri Mondor University Hospital as previously reported [5]. Briefly, an intravenous catheter was inserted into an arm and used to draw blood samples for inulin clearance measurement. The height and body weight of the patient were recorded prior to an oral water load (8 ml/kg body weight). The patient was then placed in the supine position. A priming dose of inulin (Inutest: Fresenius Pharma, Linz, Austria), 0.12 mL/kg body weight of a 25% solution diluted in 130 mL of isotonic mannitol solution, was infused intravenously (10 mL/min) into the other arm. Then a continuous infusion of Inutest 25% (0.32 mL/kg body weight) diluted in 250 mL of isotonic mannitol solution was given at a rate of 0.9 mL/min. After a 90-minute equilibration period, the bladder was emptied and urine was collected for two 30-minute periods. For inulin measurement, urine was deproteinized, the polymer was hydrolyzed with hydrochloric acid, and a colorimetric assay based on the Galli and Jeanmaire technique was performed. The first blood sample, taken before the infusions, was used as a blank. GFR was calculated as the arithmetic mean of the GFR values obtained during the two periods of urine collection. GFR-inulin data were corrected for a standard body surface area of 1.73 m^2^.

Creatinine was measured in the first blood sample, taken before the inulin infusions, using a modified Jaffe method (Randox reagent; Bayer, Montpellier, France).

In each patient the GFR was also estimated from the serum creatinine concentration by using the Cockcroft and Gault equation, as follows:

With the results multiplied by 0.85 for females; where age is in years, weight in kg and serum creatinine in micromol/L [2,3].

GFR was also estimated with a modified Cockcroft and Gault formula taking body surface area (BSA) into account, as follows [4]:

The body surface area was calculated with the Dubois and Dubois equation [13]

***The following abbreviated MDRD equation was used ***[5]

***Mayo clinic Quadratic equation ***[6] =

serum creatinine is expressed in mg/dl; if SCr < 0.8 mg/dl, use 0.8 for SCr

### Statistical analyses

As inulin clearance and GFR estimates were normally distributedin the overall population, we used repeated-measures analysis of variance with the Dunnet multiple comparisons test as post-test (Instat 3, GraphPad, San Diego USA). Correlations among the five GFR methods were studied by using the Pearson linear coefficient of correlation and coefficient of determination (Instat 3, GraphPad, San Diego USA). Paired comparisons of inulin clearance were performed with the paired t test, whereas paired comparison of creatinine used the Wilcoxon test (Instat 3, GraphPad, San Diego USA). The accuracy of the GFR estimates in the whole group was assessed in terms of the proportion of predicted values falling within 10%, 30% and 50% of the true GFR measured by inulin clearance.

In each of the two study subgroups (patients whose renal function deteriorated and those whose renal function improved), the annual slope of GFR (change in GFR in ml/min/1.73 m2/year) was used, as advocated by Fontseré and coworkers [11], to assess the variability of the prediction equations compared with the inulin method during follow-up. The annual GFR slope was determined for each GFR-inulin and with each prediction equation as the loss or gain in the glomerular filtration rate during the study period, with respect to baseline values at the start of follow-up, and standardized for 12 months [11].

As the values did not have a Gaussian distribution in the Kolmogorov and Smirnov test; [14](Instat 3, GraphPad, San Diego USA), we used the non parametric repeated-measure analysis of variance (Friedman Test) with the Dunn's multiple comparisons test as post-test to compare the five GFR methods for determining the annual slope [14](Instat 3, GraphPad, San Diego USA). P values < 0.05 were considered significant [14]. Correlations among the four GFR methods for determining the slope were studied with Spearman's rank-order correlation coefficient [14]. We then performed a concordance study as described by Bland and Altman, in which the differences between the methods are plotted against their mean values [15] (Prism 4, GraphPad, San Diego USA). The ability of a creatinine-based equation to properly categorize the trend in GFR was defined as the proportion of patients defined by the inulin clearance as having either improved or deteriorated renal function and who were correctly identified as such by the GFR estimate. The proportions of correctly categorized patients were compared by using the X^2 ^test [14] (Instat 3, GraphPad, San Diego USA). The characteristics of the patients were analyzed using either analysis of variance (parametric or non parametric according to the Gaussian distribution), a t test or the X^2 ^test. Finally, we used receiver-operator characteristic (ROC) curves to examine the ability of the estimates to discriminate progressors from improvers (Prism 4 software, Graphpad, San Diego, USA). Values in the text and tables are means ± SD or medians and ranges, depending on the normality of the distribution.

## Results

### Whole group of patients

Among the 126 patients, inulin clearance values at the start of follow-up were distributed as follows: 24% (n = 31) in stage I, 40% (n = 50) in stage II, 30% (n = 38) in stage III and 6% (n = 7) in stage IV of CKD, as defined by K-DOQI [7]. Analytical data on the whole group and subgroups are summarized in table 1.

**Table 1:** Analytical data on 126 patients with non diabetic nephropathies at baseline and at the last observation during follow-up.

	Sample Size	Age (years)	Follow-up (months)	Baseline GFR (ml/min/1.73 m^2^) (inulin clearance)	Baseline creatinine (μmol/L)	Number of GFR measurements	Final GFR (ml/min/1.73 m^2^) (inulin clearance)	Final Creatinine (μmol/L)
Whole group	126	40.36 ± 12.48 [17–69]	37.56 ± 27.04 [6–117]	71.03 ± 24.02 [20–138]	119.03 ± 50.85 [49–319]	3.43 ± 2.06 [2–11]	71.19 ± 26.93 [18–140]	123.24 ± 69.195 [53–485]

Patients with deterioration in renal function	65	39.15 ± 12.50 [20–65]	42.86 ± 29.47 [6–117]	75.30 ± 26.15 [20–138]	123.15 ± 57.85 [49–316]	3.72 ± 2.2 [2–11]	61.67 ± 25.49 [18–118]	141.81 ± 85.97 [63–485]

Patients with improvement in renal function	61	41.65 ± 12.43 [17–69]	31.91 ± 23.11 [6–93]	66.47 ± 20.78 [24–124]	114.81 ± 42.21 [53–285]	3.13 ± 1.8 [2–10]	81.32 ± 24.79 [25–140]	103.45 ± 36.31 [53–247]

Data are expressed as means ± SD [range]

Repeated-measured ANOVA showed that the differences among the five GFR methods at baseline were not due to chance (p < 0.0001). The Dunnett multiple comparison post-test showed a significant difference between inulin clearance and the standard Cockcroft and Gault estimate (t = 3.65; p < 0.01), the abbreviated MDRD equation (t = 5.59; p < 0.01), the Mayo Quadratic Equation (t = 11.83; p < 0.01), but not the BSA-modified Cockcroft and Gault estimate (t = 1.70; p > 0.05). These results are similar to those previously published for an entire cohort of 269 European adults with chronic renal disease [4].

Pearson's coefficient showed that inulin clearance correlated better with the BSA-modified Cockcroft and Gault formula (r = 0.89; 95% confidence interval: 0.84–0.92; r squared = 0.79), the Mayo Quadratic equation (r = 0.88; 95% confidence interval: 0.83–0.91; r squared = 0.77) and the abbreviated MDRD equation (r = 0.87; 95% confidence interval: 0.82–0.90; r squared = 0.75) than with the standard Cockcroft and Gault equation (r = 0.83; 95% confidence interval: 0.76–0.87; r squared = 0.69).

In the whole population the BSA-modified Cockcroft and Gault formula, the abbreviated MDRD equation and the original Cockcroft and Gault estimate were significantly more accurate than the Mayo Quadratic equation: more than 92.8% of the GFR values predicted by the BSA-modified Cockcroft and Gault formula fell within 30% of the corresponding inulin clearance values, as compared to 89.7% with the abbreviated MDRD equation, 85% with the standard Cockcroft and Gault estimate but only 67.4% with the Mayo Quadratic equation (p < 0.01 in the X^2 ^test). Accuracy is reported in Table 2.

**Table 2:** Proportion of estimated GFR values within 10% (accuracy 10%), 30% (accuracy 30%) and 50% (accuracy 50%) of the coresponding inulin clearance value.

	Accuracy 10% (95% CI)	Accuracy 30% (95% CI)	Accuracy 50% (95% CI)
Cockcroft-Gault formula	43.6% [35% – 52%]	41.3% [33% – 50%]	15% [9% – 22%]

BSA-Cockcroft Gault formula	53.2% [44% – 61%]	39.6% [31% – 48%]	7.1% [3.6% – 13%]

Abbreviated MDRD equation	37.3% [29% – 46%]	52.4% [43% – 60%]	10.3% [6% – 16,9%]

Mayo Clinic Quadratic equation	27.7% [20% – 36%]	39.7% [31% – 48%]	32.6% [24% – 41%]

Inulin clearance and creatinine values did not differ in the whole group at the end of follow-up as compared to the basal values (p > 0.05 in the t test and Wilcoxon test respectively) (Table 1). The GFR slope determined by inulin clearance for the whole group was low (-0.3 ml/min/year; range: -72 – +99) and did not differed significantly from that calculated with the GFR estimates (p > 0.05 in the Friedman test)(Cockcroft equation: – 0.12 ml/min/year; range: -60 – +102) (Cockcroft-BSA equation:

-0.18 ml/min/year; range: -60 – +109) (MDRD abbreviated equation: – 0.12 ml/min/year; range: -64 – +138) (Mayo Quadratic Equation: – 0.55 ml/min/year; range: -78 – +52). These results are explained by the fact that about half of the patients were progressors for renal failure whereas renal function improved symmetrically in the other half.

The accuracy of the prediction equations expressed as the GFR slope (ml/min/1.73 m2/year) in each of the two groups of patients (patients with a deterioration in renal function and patients with an improvement) is summarized in Tables 3 and 4.

**Table 3:** Comparison of predictive equations during follow-up in CKD patients with non-diabetic nephropathies.

	Method of GFR estimation	Slope of GFR (ml/min/1.73 m^2^/year) Median and [range]	p value in the Friedman test (non parametric ANOVA)	P value in Dunn's multiple comparison post-test
Subgroup of patients with deteriorating renal function (n = 65)	Inulin Clearance	-3.72 [-0.48 to -72]		
			
	GFR Cockcroft-Gault	-4.08 [-0.36 to -60]		NS
			
	BSA-modified Cockcroft-Gault Formula	-3.48 [-0.24 to -56]	p = 0.29	NS
			
	Abbreviated MDRD equation	-3.12 [0 to -64]		NS
			
	Mayo Clinic Quadratic equation	-3.73 [0 to -78]		NS

Subgroup of patients with improving renal function (n = 61)	Inulin Clearance	+6 [+0.36 to +99]		
			
	GFR Cockcroft-Gault	+4.2 [0 to +102]	p < 0.001	p < 0.05
			
	BSA-modified Cockcroft-Gault Formula	+3.36 [+0.12 to +109]		p < 0.05
			
	Abbreviated MDRD equation	+5.04 [0 to +138]		p < 0.05
			
	Mayo Clinic Quadratic equation	+4.74 [0 to +53]		p < 0.05

**Table 4:** Assessment of GFR changes with time using the original Cockcroft and Gault formula (CG), the BSA-modified-Cockcroft and Gault formula (BSA-CG) and the Abbreviated MDRD equation (A-MDRD), and the Mayo Clinic Quadratic equation.

		Original Cockcroft and Gault formula (CG)	BSA-modified-Cockcroft and Gault formula (BSA-CG)	Abbreviated MDRD equation (A-MDRD)	Mayo Clinic Quadratic equation (MCQ)
**Subgroup of patients with deteriorating renal function** **(n = 65)**	Spearman Correlation coefficient (95% confidence interval) with inulin clearance	r = 0.64 (0.43 – 0.78)	r = 0.67 (0.46 – 0.80)	r = 0.63 (0.41 – 0.78)	r = 0.49 (0.23 – 0.69)
	
	Patients correctly classified as having deteriorating renal function	74%	77%	75%	74%
	
	Bias (SD of bias) versus inulin clearance	0.98 (6.08)	1.37 (6.88)	1.30 (5.95)	1.32 (13.61)

**Subgroup of patients with improving renal function** **(n = 61)**	Spearman Correlation coefficient (95% confidence interval) inulin clearance	0.75 (0.58 – 0.86)	r = 0.75 (0.58 – 0.86)	r = 0.72 (0.54 – 0.84)	r = 0.46 (0.19 – 0.67)
	
	Patients correctly classified as having improving renal function	74%	74%	66%	68%
	
	Bias (SD of bias) versus inulin clearance	3.08 (7.98)	2.98 (7.63)	1.27 (8.87)	3.02 (15.46)

### Patients whose renal function deteriorated

During a mean follow-up of 42.86 months (+/- 29.47; range: 6–117), 256 determinations of GFR by inulin clearance were performed in these 65 patients and the median number of GFR per patient was 3 (range 2–11). The mean baseline GFR determined by inulin clearance was 75.30 ml/min/1.73 m2 (+/- 26.15; range: 20–138) and the mean baseline serum creatinine was 123 micromol/L (+/- 57.85; range: 49–316). In this group the median GFR slope determined by inulin clearance was – 3.72 ml/min/1.73 m2/year (range -0.48 to -72) (Tables 1 and 3).

Seventeen (26%), fifteen (23%) and sixteen (25%) of these 65 patients were wrongly classified as having no deterioration in renal function by the original Cockcroft-Gault formula and the Mayo Quadratic equation, the BSA-modified Cockcroft and the abbreviated MDRD equation respectively (Table 4). The predictive performance of the three GFR estimates for detecting a deterioration in renal function did not differ significantly (p > 0.05 in the X^2 ^test).

The Friedman test showed no difference among the five GFR methods for the determination of the GFR slope (p = 0.29) (Table 3). The Spearman rank correlation showed that inulin clearance correlated similarly with the standard Cockcroft and Gault equation (r = 0.64; 95% confidence interval: 0.43–0.78), the BSA-modified Cockcroft and Gault formula (r = 0.67; 95%; confidence interval: 0.46–0.80) and the abbreviated MDRD equation (r = 0.63; 95% confidence interval: 0.41–0.78) but less with the Mayo Quadratic equation (r = 0.49; 95% confidence interval: 0.23–0.69) (Table 4). Concordance studies with the Bland and Altman test showed a similar bias with the standard Cockcroft-Gault estimate (versus inulin clearance: mean bias = 0.98 ml/min/1.73 m2/year), the BSA-modified Cockcroft-Gault estimate (versus inulin clearance: mean bias = 1.37 ml/min/1.73 m2/year), the Abbreviated MDRD (versus inulin clearance mean bias = 1.30 ml/min/1.73 m2/year) and the Mayo Quadratic equation (versus inulin clearance mean bias = 1.32 ml/min/1.73 m2/year) (Table 4). The precision of the GFR estimates was similar with the standard Cockcroft and Gault equation (SD of bias: 6.08 ml/min/1.73 m^2^/year), the BSA-modified Cockcroft and Gault formula (SD of bias: 6.88 ml/min/1.73 m^2^/year) and the abbreviated MDRD (SD of bias: 5.95 ml/min/1.73 m^2^/year) and larger with the Mayo Quadratic equation (SD of bias: 13.61 ml/min/1.73 m2/year) (Table 4).

Forty-two of these progressor patients had 3 or more repeated GFR measures (median 5; range 3–10) and were followed-up for a median of 60 months (range: 6–117). In these patients, the Friedman test also showed no difference among the five GFR methods for the determination of the GFR slope (p > 0.05). The Spearman rank correlation showed that inulin clearance correlated better with the GFR estimates in these patients with longer follow-up: standard Cockcroft and Gault equation (r = 0.67; 95% confidence interval: 0.41–0.83); BSA-modified Cockcroft and Gault formula (r = 0.74; 95%; confidence interval: 0.52–0.87); abbreviated MDRD equation (r = 0.79; 95% confidence interval: 0.60–0.89); Mayo Quadratic equation (r = 0.71; 95% confidence interval: 0.46–0.85). Similarly, concordance studies with the Bland and Altman test showed a smaller bias with the standard Cockcroft-Gault estimate (versus inulin clearance: mean bias = 0.47 ml/min/1.73 m^2^/year), the Mayo Quadratic equation (versus inulin clearance mean bias = 0.45 ml/min/1.73 m^2^/year), and the BSA-modified Cockcroft-Gault estimate (versus inulin clearance: mean bias = 1.19 ml/min/1.73 m^2^/year), and a similar bias with the Abbreviated MDRD (versus inulin clearance mean bias = 1.43 ml/min/1.73 m^2^/year). The precision of the GFR estimates was also improved in the standard Cockcroft and Gault equation (SD of bias: 4.45 ml/min/1.73 m^2^/year) but similar in the BSA-modified Cockcroft and Gault formula (SD of bias: 6.65 ml/min/1.73 m^2^/year), the abbreviated MDRD (SD of bias: 5.98 ml/min/1.73 m^2^/year) and the Mayo Quadratic equation (SD of bias: 11.57 ml/min/1.73 m^2^/year). The improvement in the performance of these equations in patients followed-up for a longer period and having more GFR measurements strongly suggests regression toward the mean, a well-known statistical phenomenon where extreme scores regress toward the mean when remeasured [14].

### Patients with an improvement in renal function

During a mean follow-up of 31.91 months (+/- 23.11; range: 6–93) these 61 patients had 196 determinations of GFR by inulin clearance and the median number of GFR per patient was 2 (range 2–10). The mean baseline GFR determined by inulin clearance was 66.47 ml/min/1.73 m2 (+/- 20.78; range: 24–124) and the mean baseline serum creatinine was 114.81 micromol/L (+/- 42.21; range: 53–285) (Table 1). In this group, the median GFR slope determined by inulin clearance was + 6 ml/min/1.73 m^2^/year (range +0.36 to +99) (Table 1). Sixteen of these 61 patients (26%) were wrongly classified as having no improvement in renal function by the original and BSA-adjusted Cockcroft-Gault formulas; respectively 20 patients (32%) and 21 patients (34%) were wrongly classified by the Mayo Quadratic equation and the abbreviated MDRD equation (Table 4). The predictive performance of the three GFR estimates for detecting an improvement in renal function did not differ significantly (p > 0.05 in the X^2 ^test).

The Friedman test showed that the differences among the five GFR methods for the determination of the GFR slope were not due to chance (p < 0.0005)(Table 3). Dunn's multiple comparison post-test showed a significant difference between inulin clearance and the standard Cockcroft and Gault estimate (p < 0.05), the BSA-modified Cockcroft and Gault estimate (p < 0.05), the Abbreviated MDRD equation (p < 0.05) and the Mayo Quadratic Equation (p < 0.05)(Table 3). The Spearman rank correlation showed that inulin clearance correlated similarly with the standard Cockcroft and Gault equation (r = 0.75; 95% confidence interval: 0.58–0.86), the BSA-modified Cockcroft and Gault formula (r = 0.75; 95% confidence interval: 0.58–0.86), the Abbreviated MDRD equation (r = 0.72; 95% confidence interval: 0.54–0.84), but less with the Mayo Quadratic equation (r = 0.46; 95% confidence interval: 0.19–0.67) (Table 4). Concordance studies with the Bland and Altman test showed similar bias with the standard Cockcroft-Gault estimate (versus inulin clearance: mean bias = 3.08 ml/min/1.73 m^2^/year), the BSA-modified Cockcroft-Gault estimate (versus inulin clearance: mean bias = 2.98 ml/min/1.73 m^2^/year), the Mayo Quadratic equation (versus inulin clearance: mean bias = 3.02 ml/min/1.73 m^2^/year) but a smaller bias with the abbreviated MDRD (versus inulin clearance: mean bias = 1.27 ml/min/1.73 m^2^/year)(Table 4). The precision of the GFR estimates was similar with the standard Cockcroft and Gault equation (SD of bias: 7.98 ml/min/1.73 m^2^/year), the BSA-modified Cockcroft and Gault formula (SD of bias: 7.63 ml/min/1.73 m^2^/year), the abbreviated MDRD (SD of bias: 8.87 ml/min/1.73 m^2^/year) and larger for the Mayo Quadratic equation (SD of bias: 15.46 ml/min/1.73 m^2^/year) (Table 4).

Twenty-one of these patients had 3 or more repeated measures (median 5; range: 3–10) and were followed-up for a median of 65 months (range: 16–107). Interestingly, in these patients, the Friedman test also showed no difference among the five GFR methods for the determination of the GFR slope (p = 0.10). The Spearman rank correlation showed that inulin clearance also correlated better with the GFR estimates in these patients with longer follow-up; standard Cockcroft and Gault equation (r = 0.86; 95% confidence interval: 0.67–0.94); BSA-modified Cockcroft and Gault formula (r = 0.88; 95%; confidence interval: 0.72–0.95); abbreviated MDRD equation (r = 0.80; 95% confidence interval: 0.54–0.92); the Mayo Quadratic equation (r = 0.72; 95% confidence interval: 0.40–0.88). Similarly, concordance studies with the Bland and Altman test showed a smaller bias with the Abbreviated MDRD (versus inulin clearance mean bias = 0.61 ml/min/1.73 m^2^/year), the standard Cockcroft-Gault estimate (versus inulin clearance: mean bias = 2.06 ml/min/1.73 m^2^/year), and the BSA-modified Cockcroft-Gault estimate (versus inulin clearance: mean bias = 2.04 ml/min/1.73 m^2^/year) and a similar bias with the Mayo Quadratic equation (versus inulin clearance mean bias = 3.28 ml/min/1.73 m^2^/year).

The precision of the GFR estimates was also improved in the standard Cockcroft and Gault equation (SD of bias: 6.88 ml/min/1.73 m^2^/year) and the BSA-modified Cockcroft and Gault formula (SD of bias: 6.87 ml/min/1.73 m^2^/year) and similar with the abbreviated MDRD (SD of bias: 10.94 ml/min/1.73 m^2^/year) and the Mayo Quadratic equation (SD of bias: 15.98 ml/min/1.73 m^2^/year). The improvement in the performance of these equations in these improved patients followed-up for a longer period also strongly suggests a regression toward the mean [14].

### Characteristics of the patients

Progressors differed from improvers by higher proteinuria at the outset of the study (p < 0.005 Kruskal-Wallis test with Dunn's multiple comparisons test; Progressors: 1.17 g/24 h (range: 0.32–20); Improvers: 0.5 g/24 h (range: 0.10–19.70)). The percentages of patients receiving immunological treatment and drugs acting on the renin-angiotensin system were similar in the group of progressors and in the group of improvers (p > 0.05 in the X^2 ^test).

The inaccuracy of the formulae for classifying patients as improvers or progressors was not related to any of the following clinical features: age (p > 0.05 in the t test), weight (p > 0.05 in the t test), sex (p > 0.05 in the X^2 ^test), histological type of the renal disease (p > 0.05 in the X^2 ^test).

The area under the ROC curves for discriminating between progressors and improvers were very close for the 4 GFR estimates: 0.79 (95% confidence interval: 0.70–0.87) for the original Cockcroft and Gault Equation; 0.79 (95% confidence interval: 0.71–0.87) for the BSA-modified Cockcroft and Gault formula; 0.78 (95% confidence interval: 0.70–0.86) for the abbreviated MDRD equation and 0.77 (95% confidence interval: 0.68–0.85) for the Mayo Quadratic equation.

## Discussion

In 126 adults with non-diabetic chronic renal disease (mainly glomerular diseases), we estimated the time course of the glomerular filtration rate with the gold-standard method (inulin clearance). In patients with deteriorating renal function, the original Cockcroft and Gault formula, the BSA-modified Cockcroft and Gault formula, the abbreviated MDRD equation and the Mayo Quadratic equation all gave reliable estimates of the GFR slope, with an acceptable bias. In contrast in the patients with improving renal function, the original Cockcroft and Gault formula, the BSA-modified Cockcroft and Gault formula, the abbreviated MDRD equation and the Mayo Quadratic equation underestimated the gain in GFR, although this may have less important clinical consequences. In the subgroup of patients with improving renal function, the underestimation of the slope was smaller with the abbreviated MDRD equation than with the other three equations.

To date, GFR equations have been developed and validated almost exclusively with cross-sectional data sets. The Cockcroft and Gault formula is an estimate of creatinine clearance originally developed in a population of 236 Canadian patients, 209 of whom were male [2]. The Cockcroft and Gault formula was further shown to be a reliable estimate of GFR in twelve studies comparing the results of the estimate with methods giving true GFR values (inulin; 2 studies with 196 patients) or more accurate GFR values (10 studies with 1218 patients; 3 studies with 99m-Tc-DPTA; 4 studies with ^51^Cr-EDTA; 2 with iothalamate, 1 with hippuran (reviewed in [3,4]). Three studies, one published in 1984 [16] using iothalamate, an isotope with significant tubular secretion [17], the second in 1992, in a small cohort of 20 patients with type I diabetes [18] and the third comparing inulin clearance in a cohort of 269 European patients with chronic nephropathies [4], have shown that correction for body surface area (BSA) improves the accuracy of the original Cockcroft and Gault equation.

The MDRD formulas were developed as an estimate of ^125^I-Iothalamate clearance-based GFR in a population of 1628 patients with a diagnosis of CKD [5]. The MDRD equations yielded smaller median absolute errors (3.8 mL/min/1.73 m^2^) than the Cockcroft and Gault equation (6.8 mL/min/1.73 m^2^) in the princes study [11]. In recent studies conducted in France with ^51^Cr-EDTA, the MDRD equation was more accurate for the diagnosis and stratification of renal failure in diabetic type II patients [19,20]. Finally, despite the fact that the MDRD equations were developed in patients with heavily impaired renal function (CKD stages 3 and 4) [5], the abbreviated MDRD formula has been shown to properly categorize patients with CKD stage 2, and has fairly good accuracy in these patients [4,21,22].

The Mayo clinic quadratic equation is a new equation based on the results of iothalamate clearance in both 320 patients with chronic kidney diseases and 580 healthy subjects evaluated for kidney donation [6]. Elderly subjects and African-Americans were underrepresented in this sample [6]. The Mayo quadratic equation was further shown to have similar diagnostic performance to the MRDD equation in diabetic patients; in contrast to MDRD equation, the Mayo quadratic equation does not underestimate normal GFR in diabetic subjects [23];

As previously discussed, five studies, all restricted to patients with type I and II diabetes, have shown the poor accuracy of prediction equations for monitoring kidney function, unless frank renal impairment has occurred [8-12].

One study in lung transplant patients compared longitudinal follow-up based on creatinine-based formulas (Cockcroft and Gault equation and MDRD equation 7) with the Iothalamate GFR for at least 24 months, and concluded that the creatinine-based slopes correlated with Iothalamate slopes in this setting but consistently underestimated the rate of GFR decline [24]. Similar conclusions were recently drawn in study of kidney transplant patients [25]. A post-hoc analysis of the African American Study of Kidney Disease and Hypertension (AASK) has recently shown that outcomes based on the AASK creatinine formula and the MDRD equations were similar to those obtained with ^125^I-Iothalamate GFR and similarly identified most risk factors for progression of renal failure [26]. Conversely, in a cohort of 234 patients with autosomal dominant polycystic kidney disease and a baseline creatinine clearance > 70 ml/min followed for 4 years, although Iothalamate clearance, the abbreviated MDRD equation and the Cockcroft-Gault formula gave similar slopes, predictor associations for renal function decline were strongest with iothalamate clearance, because non-GFR factors (e.g. creatinine production and tubular secretion) conservatively biased associations with GFR estimates [27]. Moreover, in a retrospective cohort study of 542 subjects who had been included in the MDRD study and followed for a median of 2.6 years, the estimated GFR slope tended to underestimate measured decrements in ^125^I-Iothalamate GFR [28]. The main methodological limitations of these latter studies was the use of ^125^I-Iothalamate for GFR measurement indeed, Odlind and coworkers have shown that this isotope is subject to significant tubular secretion in chicken, rats and humans [17]. The tubular secretion of iothalamate becomes even marked in case of renal failure and can overestimate GFR by up to 34%; these authors stated that iothalamate is not an ideal reference substance for GFR determinations in clinical studies, with an accuracy comparable to that of creatinine [17].

The main limitation of our study lies in the day-to-day variations that are known to occur in inulin clearance (11%–16%), and in serum creatinine (15.5%–19.6%)[5]. Furthermore, we did not pay special attention to the calibration of serum creatinine measurements, which has been shown to be of critical importance in individuals with normal or near-normal serum creatinine values, and to influence the accuracy of MDRD equations [29-31]. In clinical trials, accurate determination of the glomerular filtration rate and correct evaluation of changes in renal function are mandatory and require direct GFR measurement with inulin, isotopes or radiocontrast media [32]. In contrast, clinicians require a less expensive and less time-consuming test than direct GFR measurement, and although the results should be accurate, they do not need to be as precise as in clinical trials. The accuracy of the creatinine-based formula for follow-up of chronic nephropathies could be improved by calibrating serum creatinine measurements (see discussion above) [29-31] and by using cimetidine combined with an enzymatic plasma creatinine assay which has been shown in a longitudinal study of type II diabetic nephropathy to abolish the discrepancies between the iothalamate slope and the original Cockcroft and Gault equation [33]. Finally, for patients at an early phase of reduction of GFR reduction (CKD stage 1 and 2) and those whose GFR is improving, because of its higher diagnostic accuracy in patients with mildly to moderately impaired kidney function, cystatin C may provide useful additional information relative to the Cockcroft and Gault formulas and MDRD equations [34,35]. Indeed, an equation combining both serum creatinine and cystatin levels with demographic and morphologic data was recently shown in children to have better accurancy than the Schwartz equation when compared to EDTA clearance [36]. Similarly, in Chinese patients with near-normal renal function, a GFR estimate combining serum creatinine and cystatin C matched DTPA-clearance more closely than MDRD equations [37]. Preliminary data strongly suggest that a combination of cystatin C and serum creatinine also improves the monitoring of kidney function in patients with diabetes mellitus [38]. Finally, in a pooled analysis of 3418 subjects with CKD of various stages living in the USA and France and evaluated for GFR by isotope clearance (iothalamate and EDTA), an equation including serum cystatin in combination with serum creatinine, age, sex and race provided a more accurate estimate of GFR than cystatin or creatinine alone [39].

## Conclusion

In patients with non diabetic nephropathies (mainly glomerular diseases) and deteriorated renal function, the original Cockcroft and Gault formula, the BSA-modified Cockcroft and Gault formula, the abbreviated MDRD equation and the Mayo Quadratic equation give reliable estimates of the GFR slope with an acceptable bias. In the subgroup of patients with an improvement in renal function, these creatinine-based formulas underestimate the gain in GFR although this may have less important clinical consequences.

## Competing interests

The authors declare that they have no competing interests.

## Authors' contributions

GR has contributed to the conception and design of the study, statistical analysis, interpreting the data, reporting of the work and wrote the article. PA supervised and performed the analysis of the glomerular filtration rate and interprétation of data. IP supervised and performed the analysis of the glomerular filtration rate and interprétation of data. MG performed the statistical analysis, tables, figures. SA has contributed to the planning, conduct and critical analysis of the report and revising of the article

## Pre-publication history

The pre-publication history for this paper can be accessed here:

http://www.biomedcentral.com/1471-2369/10/16/prepub
